# Supervising the unseen: A critical literature review on supervising students with hidden disabilities in South Africa

**DOI:** 10.4102/ajod.v15i0.1808

**Published:** 2026-01-21

**Authors:** Cheryl L. Rielander

**Affiliations:** 1 Department of Operations Management, College of Economic and Management Sciences, University of South Africa, Pretoria, South Africa

**Keywords:** hidden disabilities, higher education, invisible disabilities, students with hidden disabilities, supervision, supervisory practices

## Abstract

**Background:**

As supervisors, we often find ourselves giving a specific student more attention and leeway than others. Have you ever wondered why some students struggle more than others, even when receiving additional assistance?

**Objectives:**

This article aimed to examine the depth of supervisory knowledge and awareness in supporting students with hidden disabilities to enhance academic guidance and foster student success.

**Method:**

This article employed a systematic literature review (SLR) and thematic analysis to investigate the supervisory challenges and support mechanisms faced by students with hidden disability. The article sought to answer the research question: How does the existing literature critically address the supervision of students with hidden disabilities in higher education in supporting their academic success?

**Results:**

Students with hidden disabilities often avoid disclosing their conditions because of stigma, emotional burden and fear of being perceived as less capable. Findings revealed a significant gap in supervisory preparedness and institutional support. Supervisors, lacking adequate training and awareness, are often ill-equipped to respond effectively, leaving students feeling isolated and invalidated, and many struggle with whether their condition is ‘valid enough’ and not seen as an excuse.

**Conclusion:**

This article explored the multifaceted dimensions of supervising students with hidden disabilities, demonstrating a significant gap in supervisory preparedness, knowledge, training and awareness. The findings showed a need for targeted strategies and training programmes to equip supervisors with the necessary skills to support students with hidden disabilities and provide an inclusive supervisory environment that promotes their well-being and success.

**Contribution:**

The article offers insights into supervisory approaches for students with hidden disabilities, providing the groundwork for future empirical research and empathetic supervisory support structures.

## Introduction

Disability is a universal aspect of human life, affecting most individuals at some point in their lives. The World Health Organization (WHO) emphasises that societal attitudes towards disability have significantly evolved since the 1970s, shifting from segregation and medical treatment to inclusion and human rights (WHO 2024). This transformation has led to greater recognition of the social model of disability, highlighting how environmental and societal barriers, not just physical impairment, limit an individual’s ability to participate equally in society (Gutterman 2023:2).

The United Nations Department of Economic and Social Affairs (UNDESA) affirms that persons with disabilities possess the same fundamental rights and deserve equal opportunities as all individuals. However, their ability to fully engage in society is frequently obstructed by physical and social barriers. Additionally, the WHO emphasises that disability remains a challenging concept to define, as it is inherently complex, evolving, multifaceted, and subject to differing interpretations (Gutterman 2023:3; WHO 2024).

Disabilities are broad and complex, and as such, many types of disabilities exist ranging between mental or psychiatric, cognitive, intellectual or learning and sensory impairment (blindness or deafness, singular or combination of disabilities) (Wiltshire 2023:21). Disabled World (2025) provides an in-depth overview of invisible disabilities, which are physical, mental or neurological conditions that are not immediately apparent but significantly affect daily functioning. These include chronic illnesses such as diabetes, fibromyalgia, mental health disorders and sensory impairments not requiring visible aids. Individuals with such conditions frequently face misunderstanding, scepticism and stigma because of lack of visible symptoms, leading to under-recognition in educational and workplace settings.

Disability definitions and student classifications vary across higher education institutions (HEIs), often reflecting a dominant medical model. Support services remain fragmented, with disability units often functioning reactively and in isolation from broader inclusion strategies. Students with disabilities represent less than 2% of the total student population, and service provision is uneven. Larger units tend to support a wider range of impairments, whilst smaller ones focus mainly on visual or mobility challenges. Notably, the longevity of a dedicated support unit does not guarantee best practices (Mutanga 2017:144).

Globally, approximately one billion people are affected by disabilities, spanning physical, cognitive, sensory, psychological and learning conditions. According to Statssa.gov (2024), 1.2 million students are enrolled in educational institutions across South Africa, of whom 883 155 attend HEIs. Despite ongoing efforts to enhance accessibility for individuals with disabilities, barriers continue to exist, impeding full disclosure and participation, particularly for students with hidden disabilities.

The WHO (2023) reports that disabilities impact millions of families worldwide, highlighting persistent challenges faced by persons with disabilities in accessing equal opportunities. Wiltshire (2023:21) emphasises the broad spectrum of disabilities, underscoring the necessity for tailored approaches to meet the diverse needs of students with hidden disabilities. Recognising and addressing these disabilities is crucial, because their nonvisible nature often hides cognitive, emotional and academic challenges. Effective strategies include personalised support, awareness programmes and policy adjustments to foster an inclusive educational environment that accepts all learners, regardless of the visibility of their disabilities.

A notable gap exists in peer-reviewed literature and policy frameworks concerning disabilities, as no South African higher education policy specifically addresses hidden disabilities, with such conditions only implied. This omission is particularly significant to Sustainable Development Goal (SDG) 4, emphasising inclusive, equitable and quality education and the promotion of lifelong learning. The United Nations 2030 Agenda, specifically Section 4.7, calls for the development of knowledge and skills to support sustainable development, a sustainable lifestyle, human rights and social inclusion. In this context, human rights should consider students with hidden disabilities to ensure no student is left behind (Globalgoals.org n.d.a., n.d.b.; United Nations n.d.).

## Research methods and design

This article reflects critical engagement, methodological rigour and a novel synthesis approach via a qualitative, interpretive research design grounded in systematic literature review (SLR) principles. It explores the experiences, supervisory challenges and support mechanisms for students with hidden disabilities, using diverse academic perspectives within supervisory contexts (Lame 2019:1633).

The methodology follows the Preferred Reporting Items for Systematic Reviews and Meta-Analyses (PRISMA) framework (Page et al. 2021). A comprehensive search was conducted across Google Scholar, Scopus, Web of Science and institutional repositories, targeting peer-reviewed articles, policy papers and credible reports published between 2010 and 2025. Key search terms included supervision, hidden and/or invisible disabilities, mental health, higher education and supervisory practices. Grey literature, such as the National Development Plan 2030 and WHO Disability Fact sheets, was also included (National Planning Commission [NPC] n.d.; WHO 2023, 2024).

To reduce publication bias and enhance coverage, the search strategy was in agreement with best practices in SLR (Page et al. 2021). Citation tracking ensured the inclusion of seminal works, whilst institutional repositories provided access to policy guidelines that are often absent from peer-reviewed sources (Adams, Smart & Huff 2017; Lame 2019:1633).

Considering the heterogeneity and diversity of sources, a critical interpretive synthesis was used. Purposive sampling focussed on hidden disabilities, with sources categorised into empirical, policy, legal and developmental frameworks. Inclusion and exclusion were performed in two stages: initial screening of titles and abstracts, followed by full-text analysis against predefined criteria specific to hidden disabilities and supervision.

Utilising Dervin’s (1983) sense-making methodology, the analytical process identified contextual and theoretical gaps in supervisory practices (Munyaradzi, Arko-Achemfour & Quan-Baffour 2023; Zongozzi 2022). This approach clarified key dynamics between supervisors and students with hidden disabilities, offering insights into best practices. Reflexivity was maintained throughout, acknowledging the researcher’s positionality in interpreting findings and uncovering hidden tensions between institutional policies (Department of Higher Education and Training [DHET] 2018; Republic of South Africa [RSA] 2022) and everyday supervisory realities.

A coding framework was developed around key themes: hidden or invisible disabilities, supervision, coping strategies and institutional policies. Coding was replicated to strengthen consistency and minimise bias and to refine subthemes for clarity.

The literature review examined the nature of hidden disabilities and associated learning conditions. Core themes involved supervision, academic performance, supervisor awareness, student disclosure, coping mechanisms and perceptions of stigma. Additionally, online accessibility and distance-learning platforms were also taken into consideration. Terminologies from the South African National Policy and Strategic Frameworks were used for consistency.

The narrative synthesis of multiple academic sources facilitated a nuanced understanding of supervisory practices for students with hidden disabilities. The review focussed on peer-reviewed academic literature published between 2010 and 2025, prioritising evidence-based studies on cognitive, psychological and learning disabilities. This broader date range (2010–2024) was applied to find policy documents, institutional reports, and other grey literature essential for contextualising supervisory practices and hidden disability support. Articles that addressed hidden or invisible disabilities (e.g. attention-deficit hyperactivity disorder [ADHD], autism spectrum disorder [ASD], dyslexia, mental health conditions and chronic illnesses) within higher education or supervision contexts were included in the search criteria, as well as those that examined academic supervision, student support, disclosure or institutional responsiveness. In addition, peer-reviewed empirical studies, as well as conceptual and theoretical papers directly related to disabilities in higher education, were included.

Articles on visible disabilities, primary and secondary education or those lacking relevance to supervision were excluded. Studies that focussed on physical and visible disabilities were excluded unless they presented transferable information. Studies unrelated to the hidden disability context and those outside of the 2010–2025 range were excluded. Nonacademic sources lacking methodological rigour, non-tertiary contexts, inaccessible full texts and non-English publications were excluded from the dataset.

**FIGURE 1:**
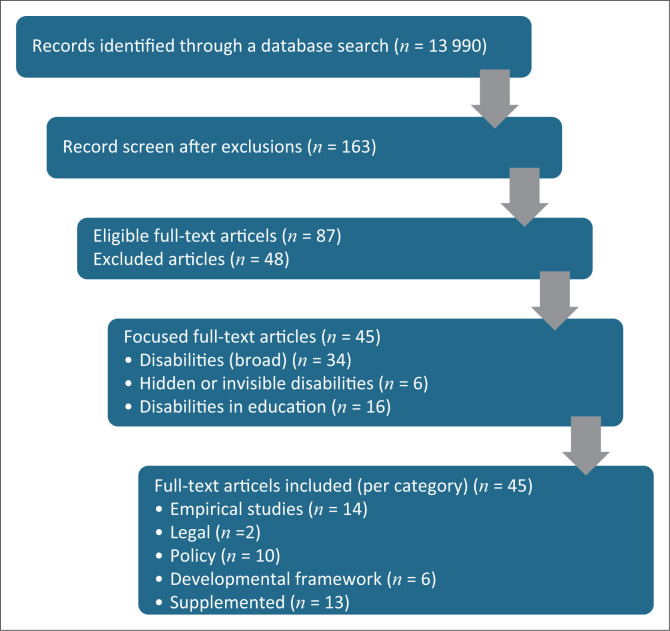
Preferred reporting items for systematic reviews and meta-analyses flow diagram.

The central research question examined whether the existing literature critically addresses the supervision of students with hidden disabilities in higher education. A total of 45 sources were included: empirical studies (*n* = 14), policy papers (*n* = 10), legal documents (*n* = 2), developmental frameworks (*n* = 6) and additional cited articles (*n* = 13). Selection was guided by relevance to hidden disabilities and supervision. The adapted PRISMA framework ensured transparency in screening and selection, as illustrated in Figure 1. The final analysis identified recurring themes, supervisory challenges, institutional responses and gaps in research and practice.

Guided by the PRISMA framework, a systematic three-stage process was employed to gather relevant academic information. The initial stage comprised conducting a comprehensive database search, focussing on scholarly articles and research studies pertinent to the topic. In addition, grey literature was reviewed, including reports and publications from reputable organisations such as the WHO, various labour organisations, and the DHET. This phase established the scope and inclusion criteria for selecting relevant sources.

The second stage centred on a thematic synthesis and coding approach. This included analysing the collected literature to identify and categorise emerging themes. Key themes involved disabilities, invisible or hidden disabilities, ADHD and dyslexia. The process aimed at revealing patterns, relationships and distinctions amongst these themes, providing a structured understanding of the subject matter.

The third stage focussed on interpreting academic articles. This involved a detailed analysis of individual interpretations, findings and conclusions related to disabilities, including invisible and hidden disabilities. The interpretation aimed to synthesise insights from various studies, highlighting commonalities and differences and providing a comprehensive understanding of the current research landscape. Overall, this structured approach facilitated a thorough exploration of the topic, ensuring that the review was systematic, transparent and grounded in credible sources.

The analysis followed a structured process: data familiarisation and then systematic coding and refinement to identify meaningful patterns whilst remaining sensitive to content nuances. This approach was guided by the sense-making framework of Mills developed in 2008, which provided a conceptual lens for examining supervisory practices (Helms Mills, Thurlow & Mills, 2010). A dedicated component of the methodology involved analysing national and institutional disability policies to assess their alignment with supervisory responsibilities. Thus, this shows a critical and previously unacknowledged policy gap, where the South African Disability Legislation and Policy Gap Analysis does not mention students with hidden disabilities, despite its explicit purpose of evaluating disability inclusion in the education sector (Centre for Augmentative and Alternative Communication [CAAC] n.d.:182). This absence is significant, as it highlights the structural gap in formal policy instruments that overlooks hidden disabilities and directly supports the research problem identified in this article. The methodological approach, therefore, addresses a core gap in the existing literature, where supervision, disability and policy implementation are typically treated as separate, nonintegrated domains (Munyaradzi et al. 2023; Zongozzi 2022). This methodological approach offers originality by addressing a gap in the current literature and legislation regarding the identification and management of students with hidden disabilities.

### Strategic policy framework on disability

In 2010, the South African President instructed the Minister of Higher Education to cultivate a skilled workforce that supports an inclusive growth trajectory encompassing persons with disabilities. The DHET affirms the rights and equitable opportunities of persons with disabilities to engage fully in the life of educational institutions (DHET 2018:18).

To advance this mandate, the Strategic Policy Framework on Disabilities was introduced to establish a postschool education and training (PSET) system tailored for persons with disabilities. This framework defines disability as a long-term physical, mental, intellectual, neurological, psychological or sensory impairment that may hinder participation in society on an equal footing with others (DHET 2018:18). The *South African Employment Equity Act No. 55 of 1998* similarly characterises disability as a long-term physical or mental impairment. South Africa’s adoption of the social definition of disability covers physical, sensory, psychological, developmental, learning, neurological and other impairments. However, many persons with disabilities, particularly students with hidden disabilities, have found this definition to be marginalising rather than enabling, limiting their development and social engagement (DHET 2018:18).

The social model of disability emphasises systemic transformation, focussing on shifting societal attitudes towards students with disabilities. It also prioritises social support mechanisms, including access to information, resources and assistance to navigate barriers (DHET 2018:19).

Despite these policy intentions, the National Transformation Agenda has acknowledged a lack of progress in the disability domain (DHET 2018:12). Institutional commitment to persons with disabilities remains inconsistent, with many technical and vocational education and training (TVET) colleges lacking formal policies and adequate support structures, particularly for students with hidden disabilities. In addition, the skill development sector fails to adequately respond to the inclusion needs of persons with disabilities, a gap especially pronounced in higher education settings (DHET 2018:13).

A 2023–2024 survey of South African public universities exhibited differences in disability support, including uneven budget allocations and fragmented service models. Institutions were predominantly categorised as either ‘Disability Targeted’ or ‘Disability Responsive’, indicating incremental progress but limited systemic transformation. Only two universities attained ‘Disability Transformative’ status. The study calls for standardised practices, refined definitions and strengthened leadership to ensure equitable inclusion for students with disabilities (Universities South Africa 2025).

The South African Disability Legislation and Policy Gap Analysis (n.d.:182), published by the University of Pretoria, dedicates only one chapter (#6) to higher education and students with disabilities, primarily addressing general disabilities and not mentioning students with hidden disabilities at all. This leaves a significant gap in the laws governing disabilities, as hidden disabilities are not mentioned or included.

### The nature of disabilities

The DHET in South Africa adheres to the legislative provisions outlined in the South African Constitution, specifically the *Bill of Rights Act. No. 108 of 1996* and the White Paper on the Rights of Persons with Disabilities of 2015. Both legislations address the right to education for persons with disabilities (De Beer et al. 2022:2); however, neither provides specific information on students with hidden disabilities. Additionally, the Disability Rights Charter of South Africa, adopted in 1992, advocated equal opportunities for persons with disabilities (SAFMH.org). With this in mind, South African HEIs need to be proactive in addressing the barriers not only for students who are disabled but also for those students with hidden disabilities.

Students with hidden disabilities have a unique set of challenges, as identified in a study conducted by Mullins and Pereyde at a Canadian University in 2013 (Mullins & Pereyde 2013:147), where they reported that hidden disabilities are seen as inferior to visible disabilities because of the legitimacy of the condition (De Beer et al. 2022:2). Many disabled students require additional effort and support compared to able-bodied or nonvisible disabled students, as they need to organise themselves and make use of effective time management, especially students with learning disabilities. This may be particularly difficult for a student who has been diagnosed with ADHD. This factor thus impacts the dropout rate of students with hidden disabilities in HEIs (De Beer et al. 2022:2; Hefiela 2024:337).

Students with hidden disabilities are prone to anxiety and depression, making adjustments to the academic environment difficult. In academia, intrinsic and extrinsic factors may complicate the journey of students with hidden disabilities; thus, the roles and responsibilities of the resource centre for students with disabilities are to support and understand the needs of students with hidden disabilities, ensuring equal opportunities for all students with disabilities, including students with hidden disabilities. Although the physical obstacles for students with disabilities may have been addressed (De Beer et al. 2022:2; Hefiela 2024:339), many of these challenges may still be prevalent for students with hidden disabilities.

Academics understand how students with hidden disabilities cope with their condition on a daily basis. This may serve as a teaching tool for teachers and academics who are unaware of this aspect (Hefiela 2024:335). Disability should be viewed as a social paradigm and not an impairment. As such, disability is a complex relationship between impairment, the social environment and students’ response to this impairment. The social model debates that disabilities result from how society is organised rather than the actual impairment. It categorises systemic barriers and negative societal attitudes that contribute to the exclusion of persons with disabilities. Although physical, sensory, and mental impairments may cause limitations, these do not result in disabilities unless society and the community fail to accommodate the differences. Academia has largely failed to integrate persons with hidden disabilities into the academic arena, thus reinforcing marginalisation (excluded from full participation and denied equitable access and opportunities). An essential aspect of the societal module is related to equality, where the module explores ways to remove barriers that restrict life choices of disabled persons and students with hidden disabilities (DHET 2018:19). This involvement rethinks supervisory practices and develops an inclusive educational environment to remove stigma and foster a sense of belonging (Munyaradzi et al. 2023; Zongozzi 2022:1649).

Additionally, disability is also linked to the medical concept that sees the disadvantages students with hidden disabilities face. As such, critical disability theory, according to Hefiela (2024:335), considers the barriers, the personal response to the impairment, and the systemic response to the invisible disability as incapable of protecting the student’s rights to be a fully functioning student within the academic arena.

### Students living with a hidden disability

In reality, students with disabilities are increasingly enrolling in tertiary institutions each year. Since the coronavirus disease 2019 (COVID-19) pandemic and the adoption of online teaching practices, academics are often unaware of students living with disabilities unless the student brings it to their attention (De Beer et al. 2022:2; Zongozzi 2022:1645).

Navigating through an academic day can be highly challenging for students with hidden disabilities, as their symptoms are not noticeable, and academics and fellow students are unaware of their inner struggle. Thus, academics and fellow students often misunderstand students with hidden disabilities, resulting in being stigmatised as difficult or lazy (De Beer et al. 2022:2). Furthermore, they fail to receive the same level of support as other impaired students, reinforcing inequalities in learning environments. A person’s impairment often becomes the ‘white elephant’ in society, functioning in an able-bodied institutional culture, shaping assumptions about capability and academic performance. Such perceptions contribute to high attrition and failure rates for students with disabilities (Munyaradzi et al. 2023:110).

Hefiela’s (2024:337) critical disability theory focusses on human rights across diverse cultures, where disabilities are recognised in all of them. As such, an intersectional framing of disability that incorporates culture and identity is essential, particularly for hidden disabilities that remain overlooked (Hefiela 2024:337).

A strategic policy framework on disability for the postschool education system emphasises that persons with disabilities have the same rights and privileges as every citizen, enabling them to participate fully in society. Regrettably, the marginalisation of disabled persons is still prevalent in our society. The strategic policy framework on disability for the postschool education system was developed to standardise a supportive environment for students with disabilities; however, barriers and prejudices persist in our educational institutions (Munyaradzi et al. 2023:106).

Despite accessibility initiatives in HEIs, students with hidden disabilities face unique challenges and remain under-represented, as access is often focussed on physical accessibility (Munyaradzi et al. 2023:110). Facilitating access is complicated, where attention is generally given to access points for students who are disabled, assuming that once a person with a disability is accepted into the higher education institutional system, the process is complete. This is not the case; it is more complicated than just ensuring access for students with disabilities, and it is a multilayered approach that should address inclusive and accessible education for persons with disabilities (Hefiela 2024:337).

A study conducted by Munyaradzi et al. (2023:116) reported that students with disabilities found integrating into the tertiary educational environment difficult and challenging, and many suggested that the physical environment did not accommodate their disabilities. This study reports that students with disabilities continue to be discriminated. If students living with disabilities report these challenges, what support is given to students with hidden disabilities in tertiary institutions? Furthermore, the study by Munyaradzi et al. (2023:106) reported several obstacles that prevented students from utilising student support services. Additionally, policies on support for students with disabilities were found to be nonexistent as stated in the study by Munyaradzi et al. (2023:117).

Students with hidden disabilities undeniably face unique challenges in higher institutions, as they navigate academic and social environments with limited support. Key points, as identified by Mahomed (2020) and De Beer et al. (2022:2), are as follows:

- Identity and perception problems, such as fear of being stigmatised, can discourage students from disclosing their disability.
- Academic challenges in the form of a chronic illness can reduce their available study time. Disabilities such as ADHD can affect the time management and cognitive functions of a student, leading to academic underperformance.
- Social and organisational barriers, such as poor social understanding of unwritten social rules, can create a barrier for a student with Asperger’s syndrome.
- One-dimensional learning models and a failure to accommodate diverse learning needs limit student success.
- A lack of senior leadership and under-resourced disability services hinder inclusive education.

Addressing these barriers requires tailored support, inclusive practices, and a deeper understanding of the nuanced struggles faced by students with hidden disabilities (Hefiela 2024:333–334).

### Inclusive and accessible education

In South Africa, inclusive and accessible education is guided by the *Higher Education Act No. 101 of 1997* and the national plan for PSET (2021–2030) that anticipates establishing quality educational opportunities for students. In addition, the plan aims to support postschool sector institutions more effectively, enabling them to enhance the quality of their education (RSA 2022:12).

For many students, the decision to pursue tertiary education may present its own challenges, such as prioritising medical care over education, instructional accessibility and disability support. Students with hidden disabilities struggle to cope with their impairment, impacting their educational achievement as they may undervalue their academic abilities because of stigmatisation, leading to poor academic performance (Mullins & Pereyde 2013:147). The fluctuating nature of the student’s condition affects the student’s ability to progress and accomplish their academic journey (Hefiela 2024:338).

Students with hidden disabilities ought to be allowed access to quality higher education and support. Comprehensive open-distance and e-learning (CODeL) institutions are better positioned to improve quality and increase diversity, given their large student bodies, including students with hidden disabilities. This has the potential to produce a skilled workforce, regardless of the disability (Zongozzi 2022:1646–1647). According to Zongozzi (2022:1647), quality in higher education is misunderstood and misinterpreted, especially in South Africa. Despite operational experience, Zongozzi (2022:1647) states that Van Damm (2002:43) found that academic quality has different meanings for different people.

### Supervising students with hidden disabilities

Most supervisors lack awareness and training related to hidden disabilities and how to manage students with disabilities. Students often report being misunderstood and unsupported, particularly students with hidden disabilities (Couzens et al. 2015:24; Zongozzi 2022:1647). Students with hidden disabilities report experiencing isolation, leading to anxiety and often burnout, because of their condition and constantly having to navigate within an unsupported academic environment. In the absence of adequate student support, students often develop coping mechanisms to manage their academic challenges. They may also revert to peer networks and personal resilience to navigate their academic journey (De Beer et al. 2022:2; Hefiela 2024:338).

Coping methods for students with hidden disabilities are behaviours individuals use to adapt to their environment and meet their personal needs to address challenges in an academic setting. The International Finance Corporation (IFC) has published a report entitled Investing in inclusion: A guide to disability-inclusive education for HEIs (2024) which explains that students with learning disabilities often struggle with distractions, a lack of support, and frustration, leading them to develop adaptive coping mechanisms, such as dropping challenging subjects, extra preparation, and using tools like visual diagrams and assistive Android-based technologies. Coping methods may involve taking additional rest breaks, performing meditation, reflecting on triggers and improving student–staff interactions. In addition, cognitive avoidance is common amongst students with learning disabilities, requiring stress management, such as behaviour adjustment and improved academic interaction (Hefiela 2024:338; IFC 2024). Students with hidden disabilities must not be discriminated against based on laziness or inability to pass. Academics be aware of maladaptive coping of students, including suppression, substance abuse, and problem drinking, which may escalate mental health issues (Kline & Davidson 2022:129). Furthermore, interpersonal skills or perception management may help address academic attitudes towards students with disabilities, thereby promoting adaptive coping strategies such as acceptance, cognitive reappraisal, problem-solving, discussing emotional distress and stress moderation to prevent symptom escalation (Hardini, Tarigan & Trang 2022:7; Hefiela 2024:337–338).

Algorani and Gupta (2023:1) describe coping behaviour for students with hidden disabilities as conscious strategies used to manage stress, distinct from unconscious defence mechanisms. This includes reactive and proactive approaches, as well as four key types: problem-focussed, emotion-focussed, meaning-focussed and social coping (Folkman & Moskowitz 2004). These range from solving problems and regulating emotions to finding meaning and seeking support from others. Students with disabilities use diverse strategies to succeed academically, including autonomous learning techniques, time management and digital tools (Lo & Yuen 2017), as well as notetaking and asking for assistance (Richardson 2021:132).

Raising awareness about the rights of people with disabilities is vital, as emphasised in Article 8 of the Convention on the Rights of Persons with Disabilities (United Nations 2006). Furthermore, it strives to combat stereotypes, prejudices and harmful practices concerning persons with disabilities. Similarly, the strategic policy framework on disability for the PSET system highlights the need for greater academic awareness of disability issues and responsive support for students with hidden disabilities (DHET 2018). Despite this, many academics lack awareness of students with hidden disabilities in their institutions as indicated by responses in a study conducted by Zongozzi (2022:1651) in a South African Open Distance and e-Learning (OdeL) institution, where participants stated that no student had informed them of a disability, and were uncertain if the university should notify lecturers about students with disabilities. Participants were unsure if the university had a dedicated disability support unit for students with disabilities. Furthermore, academics reported not having encountered students with disabilities and not preparing any special study materials or exams for them (Hefiela 2024:338; Zongozzi 2022:1651).

The lack of awareness and processes to identify students with disabilities signifies these students often go unnoticed, resulting in inadequate support. This can lead to perceptions of incompetence or failure when students with disabilities struggle academically, which may result in stigmatisation. In addition, repeated failure undermines their self-esteem, leading to disengagement, mediocre performance and eventual dropout (Hefiela 2024:338; Zongozzi 2022:1651).

However, feedback from academics shows significant gaps in accommodating students with disabilities, including those with hidden disabilities, as noted in a study by Zongozzi (2022:1651–1652). In this study, as per participants, existing text-based modules exclude blind students and should involve upgrading content delivery systems. Moreover, reliance on printed study materials challenges students with visual, hearing and mobility impairments, as it is assumed that students can cope. Some academics admit to neglecting the adaptation of learning materials for students with disabilities (Pudaruth, Gunputh & Singh 2017:3; Zongozzi 2022:1652). As alluded to, most supervisors lack awareness and training related to hidden disabilities. Furthermore, supervisors need to develop empathy and flexibility to build student trust and ensure psychological support in classrooms (Tillotson et al. 2023). They should offer effective communication channels and actively listen to students’ challenges and experiences and also need to be flexible with deadlines, taking into account the student’s disability and capacity (Hadjikakou & Hartas 2008:103; Zongozzi 2022:1652).

Academics and supervisors should consider adapting their educational practices to enhance the learning of students with disabilities, especially students with hidden disabilities. Adopting e-learning strategies is flexible, providing students with hidden disabilities access to tertiary studies without them being marginalised and left behind (Pudaruth et al. 2017:3).

This affirms the ongoing challenges in implementing disability strategies in higher education, despite policy shifts. It underscores the importance of ensuring welcoming educational environments with inclusive lecturers, practical and effective tools and tailored learning strategies (Zongozzi 2022:1652). The 2018 Strategic Policy Framework on Disability for the PSET System calls for integrating universal design into curricula to benefit all students. Whilst professional development initiatives grow globally, the policy places responsibility on academic institutions. Consequently, inaccessible learning materials fail to meet quality criteria, as they lack fitness for purpose, value for money and transformative elements (DHET 2018:7; Zongozzi 2022:1652).

To combat the achievement gap between able-bodied and students with hidden disabilities, effective teaching, communication and presence by academics, through emotional attunement and validation of the student, the support for emotional regulation provided to the student when needed and the supervisor’s own professional development and responsiveness (Inclusive Teach 2025) are essential for quality education for students. Academics have expressed discomfort and uncertainty in teaching students with disabilities, mainly as a result of a lack of knowledge, preparedness and training. In a study by Zongozzi (2022:1653), an example was shared of limited accommodation for an autistic student, as the focus was on content rather than language during script marking. Academics are of the opinion that challenges such as concentration disorders are beyond their ability to address because of the specialisation of the disorder, as well as being unprepared to manage students with disabilities or students with hidden disabilities (De Beer et al. 2022:2). This may lead to a perceived lack of support amongst students (Zongozzi 2022:1653).

This affirms ongoing challenges in implementing disability strategies in higher education, despite shifts in policy post-apartheid (Zongozzi 2022:1652). Leading universities in South Africa, despite having strong disability policies, often struggle to implement them. As HEIs are solely dependent on their centralised disability unit or department, as described by Universities South Africa in its report to the Transformation Managers’ Forum in March 2025, and on lecturers to manage students with disabilities, as well as those with hidden disabilities (Zongozzi 2022:1653). The centralised disability unit may provide support services in sign language, braille and software for use by individuals with disabilities. The centralised disability unit at each public university in South Africa requires developing institutional policies to support its processes (Pudaruth et al. 2017:4; Universities South Africa 2025).

Disability policy implementation requires translating organisational goals and objectives to support students with disabilities and students with hidden disabilities (Zongozzi 2022:1652). However, challenges exist because of a lack of commitment, capacity, clear objectives and resources amongst implementers (Adjei-Amoako 2016:870). Key stakeholders, including students with disabilities and staff, do not actively involve in policy implementation, missing a valuable opportunity to contribute to advocacy and expertise (White & Summers 2017:82; DHET 2018). As such, obtaining commitment from all levels is essential, as policy weaknesses are evident in implementation and staff training for inclusive education. Consequently, strong institutional commitment and systematic training at all levels are essential, as persistent gaps in policy implementation and staff preparedness continue to challenge the realisation of inclusive education (Munyaradzi et al. 2023:117; Pudaruth et al. 2017:4). Without proper support and participation, the inclusion policy will fail to make its intended impact (Zongozzi 2022:1652).

Furthermore, supervisors must listen attentively to their students, as students may provide subconscious/hidden clues regarding their hidden disabilities and the challenges they face (Pudaruth et al. 2017:3). Supervision is not just about the supervisor’s technical and supervisory skills; it is also about how they connect, communicate, and support their students. Supervisors and students must build a strong, mutually trusting, supportive, and trusting relationship (Couzens et al. 2015:24).

### Conceptual supervisory framework

**FIGURE 2:**
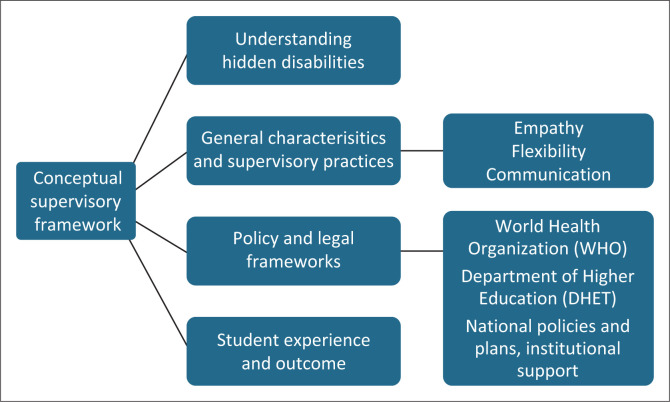
Foundation for a conceptual framework for inclusive supervision of students with hidden disabilities.

Utilising key themes identified in the literature and synthesising supervisory, institutional and student perspectives, a conceptual framework was developed to illustrate the interconnected factors influencing inclusive supervision practices. Figure 2 provides a foundation for a conceptual framework that maps student experiences, supervisory practices and institutional policy development to accommodate and support students with hidden disabilities. This framework maps the synthesis of the thematic findings to provide an explanatory tool to guide inclusive supervision.

**FIGURE 3:**
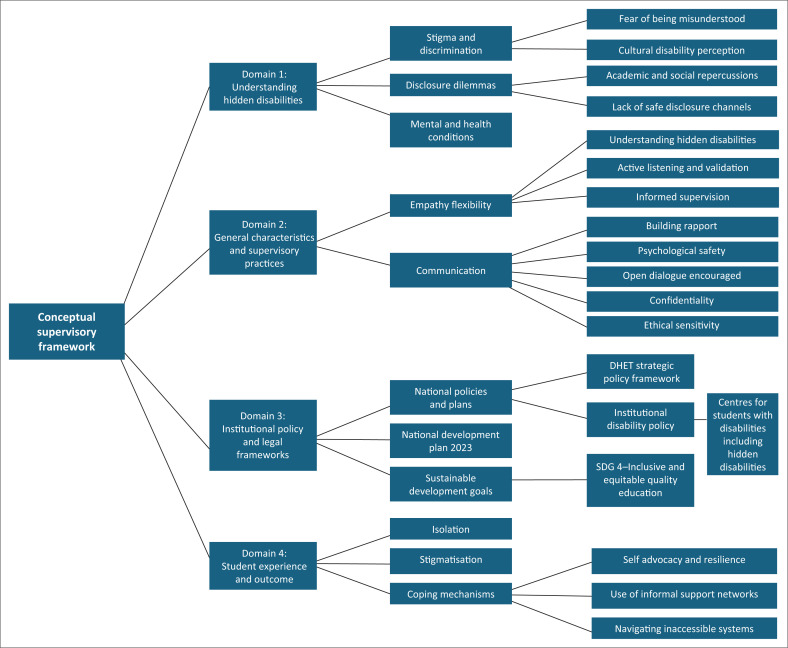
Framework for inclusive supervision of students with hidden disabilities.

Building on the foundations established in Figure 2, the conceptual framework (Figure 3) extends the discussion by structuring the synthesis of findings derived from the SLR. This expanded framework integrates heterogeneous sources, including empirical studies, policy documents and global development frameworks, to provide a more comprehensive understanding of inclusive supervision. The framework organises the literature into four core domains, each representing a key dimension that influences supervision in support of students with hidden disabilities:

- **Understanding hidden disabiliti es in higher education:** This domain focusses on the invisible (hidden) aspects of disabilities that significantly impact a student’s academic journey, cognitive processes and emotional well-being. Through the identification of aspects not immediately visible to the supervisor, but which affect the student’s learning, cognitive and emotional well-being. As such, students often hesitate to disclose these conditions because of stigma, discrimination and lack of trust. A key challenge within this domain is the reluctance of students to reveal their hidden disabilities and their experiences, which are often focussed on lived realities.
- **Supervisory practices** (roles, responsibilities and communication): This domain emphasises the importance of communication and trust between the supervisor and the student. Additionally, qualities such as empathy and understanding, flexibility, open and honest communication, flexibility and adaptability and listening skills are crucial. Lack of knowledge and training related to disabilities often plays a vital role.
- **Institutional policies and legal frameworks:** This domain explores the crucial link between national policies, institutional support, and the lived experiences of students with hidden disabilities. The gap and disconnect between institutional policies and legal frameworks, such as national policies and plans, and institutional support.
- **Student experiences and outcomes:** This domain focusses on the lived experiences of students with hidden disabilities and the impact of supervision on their academic outcomes. Students with hidden disabilities often face unique challenges, such as isolation, stigmatisation, disclosure and coping strategies used to navigate supervision challenges.

This framework (Figure 3) aims to inform supervisory practices that respond to the needs of students with hidden disabilities in higher education. It reveals the intricacies of supervising students with hidden disabilities and emphasises the need for supervisor empathy, flexibility and aggressive institutional support. Therefore, fostering a climate of trust and understanding is paramount. Supervisors need to be aware of the potential for hidden disabilities and create an environment where students feel safe and supported.

### Ethical considerations

Although this study relies on secondary sources, the researcher maintained ethical rigour by ensuring accurate representation of the original work, proper citation and respect for contexts in which the primary research was conducted. Reflexivity was applied to remain conscious of the researcher’s positionality and potential biases.

A project was registered with the University of South Africa (UNISA) College of Economic and Management Sciences Research Ethics Committee in November 2024, focussing on persons with hidden or invisible disabilities and special needs. Approval was granted in December 2024 under reference number 5948, and all ethical considerations were followed in this conceptual article.

To mitigate selection bias, the article prioritised peer-reviewed sources across major databases, with systematic screening and thematic categorisation to manage potential researcher bias.

## Results and discussion

Guided by the PRISMA framework, the evidence base covered 2010–2025 literature, where the overall flow of screening and selection is summarised in Figure 1. These findings were further interpreted through the Conceptual Supervisory Framework (Figures 2 and 3).

It is noted across contexts that students with hidden disabilities report reluctance to disclose due to anticipated stigma, fear of being perceived as incompetent, and concerns about fairness or being seen as “making excuses” (De Beer et al. 2022; Mullins & Pereyde 2013). Qualitative accounts show how nondisclosure is shaped by identity threats and social interpretation, often intensifying anxiety, isolation, and academic under-performance (Kline & Davidson 2022; Hefiela 2024). In South Africa, these risks are compounded by uneven institutional awareness and the dominance of able-bodied norms in teaching and assessment environments, which can invalidate lived experiences. The visibility gap thus produces doubt about the legitimacy of need and erodes trust between students and academic staff (Mullins & Pereyde 2013; De Beer et al. 2022).

In response to the research question, does existing literature critically address the supervision of students with hidden disabilities in higher education in supporting the academic success of students with hidden disabilities? The literature highlighted the challenges tertiary institutions face in incorporating basic disabilities as a critical component of diversity. The article highlights the gaps and practical implications of the often-misunderstood hidden disabilities and lack of correct supervisory attention. With inadequate institutional support and effective intervention, students with hidden disabilities face discrimination, marginalisation, and victimisation, leading to underperformance.

More strategies need to be explored to address the complex aspects of students living with hidden disabilities to ensure the creation of an inclusive and supportive learning environment for students with hidden disabilities. This remains elusive if supervisors continue to distance themselves from providing a supportive learning environment for these unique students. Simply transferring responsibility to disability support units limits and frustrates the learning of these students (Pudaruth et al. 2017:3). Supervisors need to be proactive in managing students with hidden disabilities. Thus, more formal scientific research is required to document both the supervisors’ and the students’ experiences with hidden disabilities.

## Conclusion

Some perceive disability as a form of inequality that affects self-esteem and belonging. Perceptions are often shaped by societal norms that prioritise visible abilities and productivity, marginalising those with nonvisible conditions. When students with hidden disabilities face environments lacking empathy, flexibility and support, they experience feelings of inadequacy and exclusion, ultimately affecting their academic performance, creating a perception of laziness or of being a difficult student. Eventually, this will not only affect their academic performance but also their emotional well-being. Understanding their challenges and providing practical support grounded in awareness, inclusive supervision and informed policy practices are essential to foster an academic environment that allows students with hidden disabilities to thrive with confidence.

This article presents a conceptual framework for inclusive supervision in higher education, synthesising diverse sources. The article highlights the importance of addressing hidden disabilities through informed, empathetic and policy-aligned supervisory practices that recognise the unique challenges students with disabilities face. Whilst there is growing recognition of hidden disabilities in higher education, the literature does not yet comprehensively address the supervision of students with hidden disabilities in a way that fully supports their academic success.

There are, however, limitations that should be acknowledged. Firstly, the article draws solely on secondary data, which does not capture the nuances of student–supervisor dynamics. Secondly, the reliance on higher education literature may exclude valuable regional and context-specific information from other areas such as secondary and vocational education and workplace training. Lastly, the article focussed on supervisory practices and did not explore other aspects, such as logistical and technological accessibility.

Future research should focus on primary and empirical studies to capture the voices of students with hidden disabilities, as well as those of their supervisors, to provide more contextualised data. In addition, future studies should investigate digital and distance-learning supervision to ensure inclusive practices across both the physical and virtual academic environments.

### Contribution

Based on a critical literature review, this article contributes to the growing dialogue surrounding the often-overlooked experiences of students with hidden disabilities. This synthesises diverse empirical studies, policy documents, legal frameworks and developmental models to offer a holistic understanding of supervisory challenges and opportunities. By applying the PRISMA framework, the study ensures transparency and rigour. The inclusion of diverse documents broadens the investigation beyond academic literature, enriching the analysis with perspectives from policy and practice.

By integrating existing literature and identifying gaps, the article provides an understanding of the challenges faced by students with hidden disabilities and how they are (or are not) supported in an academic environment. It provides insight into supervisory approaches that can unintentionally exclude students with hidden disabilities. The insights from this article can inform curriculum development, supervisor training programmes and institutional policy reforms aimed at fostering inclusive academic environments. It lays the groundwork for future empirical research on students with hidden disabilities and the development of ethically sound, empathetic supervisory support structures.
